# Food Ingredients Involved in White-to-Brown Adipose Tissue Conversion and in Calorie Burning

**DOI:** 10.3389/fphys.2018.01954

**Published:** 2019-01-11

**Authors:** Hamza El Hadi, Angelo Di Vincenzo, Roberto Vettor, Marco Rossato

**Affiliations:** Internal Medicine 3, Department of Medicine, University of Padua, Padua, Italy

**Keywords:** brown adipose tissue, capsaicin, resveratrol, green tea, curcumin, menthol, Omega-3 polyunsaturated fatty acids

## Abstract

Obesity is the consequence of chronic positive energy balance and considered a leading risk factor for cardiovascular and metabolic diseases. Due to its epidemic trends among children and adults, there is an increasing interest in implementing new therapeutic interventions to tackle overweight and obesity. Activation of brown adipose tissue (BAT) represents today a promising strategy to enhance energy expenditure (EE) through heat production. More recently, “browning” of white adipose tissue (WAT) has gained increasing attention in research area as an alternative method in stimulating energy dissipation. This minireview aims to summarize the current knowledge of some dietary compounds that have been shown to promote BAT activation and WAT browning with subsequent beneficial health effects.

## Introduction

Obesity is defined as an abnormal body fat accumulation due to chronic periods of imbalance between energy intake and expenditure. A major concern is the high risk of accompanying cluster of comorbidities, such as CVDs, metabolic syndrome and some forms of cancer (Andolfi and Fisichella, 2018).

The management of body weight is mainly based on lifestyle modifications and modulating the absorption of food. On the other hand, approaches aimed to enhance EE represent an alternative tool for counteracting obesity and related cardiometabolic disorders (Khera et al., 2016).

In addition to the well known WAT storing lipids and undergoing pathological structural and functional changes during obesity, mammals including adult humans are also equipped with BAT conferred with thermogenic capacity called adaptive or NST (Virtanen et al., 2009; Rosenwald and Wolfrum, 2014). BAT is a metabolically active tissue rich in mitochondria containing UCP1 that mediates the uncoupling of electron transport which leads to a decrease in the generation of ATP from adenosine diphosphate (ADP) with subsequent heat production (Bartelt and Heeren, 2014; Rossato et al., 2014). Today, the induction of BAT thermogenesis represents a promising protective strategy to combat obesity in humans by increasing EE (Rosenwald and Wolfrum, 2014).

**FIGURE 1:**
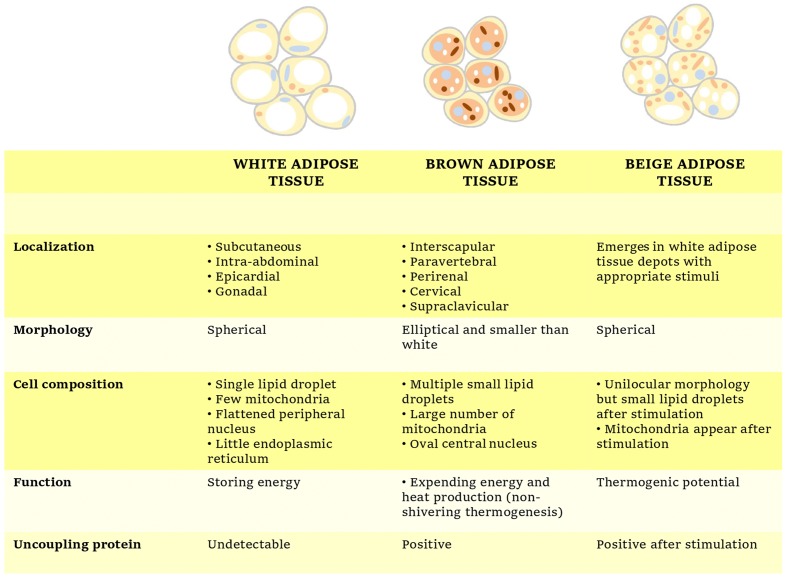
Overview of the main characteristics of white, brown, and beige adipocytes.

However, NST is not restricted only to classical brown adipocytes since certain stimulations as cold exposure or ADRB3 activators can cause the so-called beige or brown-like adipocytes to emerge within WAT depots in a process termed “WAT browning” (Kiefer, 2017). The main physiological, biochemical and morphological characteristics of each adipose tissue subtype have been briefly summarized in Figure 1.

Research over the last decades has provided evidence to support the role of bioactive dietary components in the prevention and/or treatment of obesity and associated metabolic disorders (Martínez-González et al., 2015; Amiot et al., 2016). The current minireview aims to summarize the latest findings concerning the activation of BAT or browning of WAT induced by specific dietary components, including capsaicin, resveratrol, curcumin, green tea, menthol and fish-derived Omega-3 fatty acids.

**FIGURE 2:**
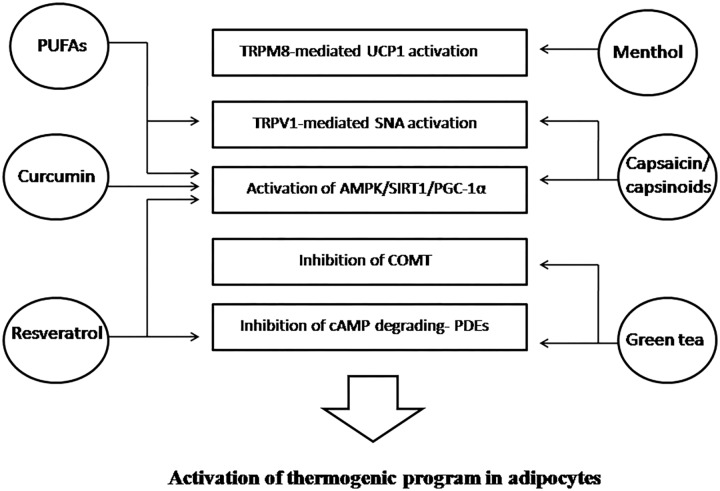
Food-derived components stimulating energy expenditure and their mechanisms of action involved in the activation of BAT or in the induction of WAT browning. BAT, brown adipose tissue; WAT, white adipose tissue; TRPM8, transient receptor potential cation channel melastatin 8; UCP1, uncoupling protein 1; TRPV1, transient receptor potential vanilloid 1; SNA, sympathetic nerve activity; AMPK 5′, adenosine monophosphate-activated protein kinase, SIRT1, sirtuin-1; PGC-1α, peroxisome proliferator-activated receptor gamma coactivator 1-alpha; COMT, catechol-O-methyl-transferase cAMP; cyclic adenosine monophosphate; PDEs, phosphodiesterases; PUFAs, polyunsaturated fatty acids.

**FIGURE 3:**
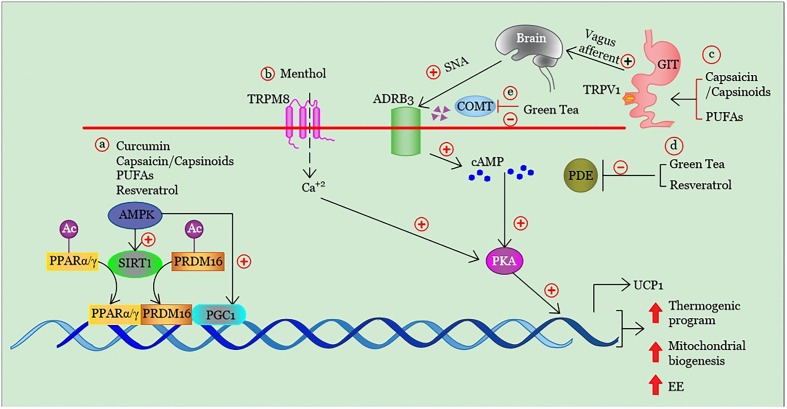
Summary of the mechanisms involved in the stimulation of brown adipogenesis, mitochondrial biogenesis and energy expenditure by some dietary molecules. **(a)** The direct and/ or indirect (via AMPK) activation of SIRT1 induces deacetylation and interaction of key transcription factors promoting brown and beige adipogenesis as PPARα/γ and PRDM16. The PPAR/PRDM16 complex is also able to bind and activate PGC1α, another cofactor specifically expressed in brown and beige adipocytes that stimulates the transcription of several genes involved in thermogenesis and mitochondrial biogenesis. Similarly, AMPK can also directly enhance PGC1α activity by phosphorylation, thus increasing mitochondrial biogenesis. **(b)** TRPM8 activation in brown adipocytes enhances the expression of thermogenic genes via Ca^2+^-dependent PKA signaling pathway. **(c)** Activation TRPV1 receptors in GIT, and consequent stimulation of the vagal afferent pathways leads to activation of neurons within the ventromedial hypothalamus. This mechanism of action induces a cold-independent adrenergic response that mediates brown adipogenesis. The adrenergic stimulation in brown adipocytes can be also promoted by the reduction of degradation of **(d)** cAMP and **(e)** norepinephrine through direct inhibition of PDEs and COMT activity, respectively. TRPM8, transient receptor potential cation channel melastatin 8; UCP1, uncoupling protein 1; TRPV1, transient receptor potential vanilloid 1; SNA, sympathetic nerve activity; AMPK, adenosine monophosphate-activated protein kinase, SIRT1, sirtuin-1; PGC-1α, peroxisome proliferator-activated receptor gamma coactivator 1-alpha; COMT, catechol-O-methyl-transferase cAMP; cyclic adenosine monophosphate; PDEs, phosphodiesterases; PUFAs, polyunsaturated fatty acids; Ac, acetyl group; cAMP, cyclic adenosine monophosphate; EE, energy expenditure; PPARα/γ peroxisome proliferator-activated receptor alpha/gamma; PKA, protein kinase A, PRDM16; PR-domain containing 16, (+), stimulation; (–), inhibition; ↑, increase.

In Figures 2 and 3 we have summarized the main physiologic mechanisms involved in BAT promotion through these dietary compounds.

## Capsaicin and Capsinoids

Capsaicin (8-methyl-N-vanillyl-6-nonenamide) is an active component of hot pepper and belongs to the capsicum genus of plants. This alkaloid is responsible for the pungency and hotness sensation of chili peppers (Thiele et al., 2008). Capsinoids, which include dihydrocapsiate, nordihydrocapsiate, and capsiate, have the same chemical structure as capsaicin. They are active ingredients found in the non-pungent type of red chili (Kobata et al., 1999). Both capsaicin and capsinoids have elicited enormous interest due to their role in enhancing fat oxidation and EE (Ludy et al., 2012). The oral treatment with capsinoids (6 mg/kg for 12 weeks) in overweight or obese subjects was associated with abdominal fat loss and increase in fat oxidation compared with placebo group (Snitker et al., 2009). Moreover, Lejeune et al. (2003) showed that capsaicin treatment (135 mg per day) in overweight subjects induced elevation of the resting EE. In another randomized placebo-controlled study, capsinoids (10 mg/kg per day) were able to increase EE, VO_2_, and enhance fat burning (Inoue et al., 2007). The thermogenic and anti-obesity effects of capsaicin and capsinoids have been shown to be mediated, at least in part, by the promotion of BAT activity. Yoneshiro et al. (2012) assessed the short-term effects of dietary supplementation of capsinoids (9 mg) on EE and BAT activity in healthy males. BAT activity was assessed by [^18^F]- FDG-PET. The authors demonstrated a significant increase of EE (by 15.2 kJ/h) in subjects with active BAT. On the other hand, only a slight increase in EE (by 1.7 kJ/h) after oral administration of capsinoids was observed in the BAT-negative group (Yoneshiro et al., 2012).

The intragastric administration of capsaicin analog in rats induced an increase in SNA of BAT via activation of TRPV1 channels expressed along the gastrointestinal tract (Ono et al., 2011). These findings were supported by another study that showed a TRPV1-dependent increase in the temperature of the colon and intrascapular BAT after jejunal administration of capsinoids in mice. This effect was inhibited by an extrinsic denervation of the jejunal segment suggesting a role of the gastrointestinal vagus nerve in capsinoids-mediated thermogenesis (Kawabata et al., 2009). In a recent report, a combination of mild cold exposure and capsinoids in C57BL/6 mice has been shown to promote the development of both brown and beige adipocytes in inguinal WAT suggesting a centrally mediated effect of capsinoids. Capsinoids interact with TRPV1 receptors in gut, which in turn stimulate the vagal afferent pathways leading to activation of neurons within the ventromedial hypothalamus. This mechanism of action induces a cold-independent adrenergic response in WAT, leading to an increase in PRDM16 levels and stability. Therefore, the two β-adrenergic pathways mediate synergically the capsinoids-induced beige adipocyte biogenesis (Ohyama et al., 2016).

Moreover, Baskaran et al. (2016) showed that capsaicin triggered browning of WAT by promoting the expression of SIRT1, UCP1, BMP8B, and PPARγ, PGC-1α in white adipocytes. SIRT1 is a NAD^+^- dependent deacetylase of several transcription factors including PGC-1α, hence modulating oxidative mitochondrial metabolism (Cantó and Auwerx, 2009). In white adipocytes, SIRT1 also induces deacetylation and interaction of PPARγ and PR-domanin containing 16 (PRDM16) and thereby regulates key factors involved in BAT development (Qiang et al., 2012). Despite the fact that the direct mechanism by which capsaicin and its derivatives activate BAT has been demonstrated in animal models, much work is still to be done in humans.

## Resveratrol

Resveratrol (trans-3,5,4′-trihydroxystilben) is a natural polyphenol that has attracted a lot of research interests mainly in the field of obesity management (Kim and Park, 2011). This compound was first found in the roots of white hellebore, and then in mulberries, red wine, grapes and peanuts (Burns et al., 2002).

The administration of resveratrol (400 mg/kg/day for 10 weeks) in mice fed with HFD has shown to reduce visceral fat-pad weights and suppress adipogenesis in epididymal white adipocytes (Kim et al., 2011). Furthermore, Rayalam et al., (2008) provided additional evidence for the inhibitory effects of resveratrol on adipogenesis. Resveratrol reduced adipocytes viability and downregulated the expression of adipogenic factors such as SREBP-1c, LPL, C/EBP-α, HSL, and PPARγ. In addition, resveratrol upregulated genes involved in mitochondrial biogenesis such as mitofusin (Mfn)-2 and UCP1 (Rayalam et al., 2008).

Low concentrations of resveratrol have been shown to promote the expression of brown adipogenic markers including UCP1, PRDM16, PGC1α and CIDEA in stromal vascular cells isolated from mouse inguinal WAT, suggesting a main role of resveratrol in WAT browning (Wang et al., 2015a). Similar findings were observed in stromal primary vascular cells separated from interscapular BAT after treatment by resveratrol in vitro (Wang et al., 2017). The browning effect of resveratrol has been suggested to be mediated by 5′ adenosine monophosphate-activated protein kinase (AMPK) α1 activation, since these changes wereabsent in cells lacking AMPKα1 (Wang et al., 2015a, 2017).

In C57BL/6J mice, the ingestion of HFD supplemented with resveratrol for 15 weeks led to an increase in adaptive thermogenesis in response to external cooling. Moreover, BAT of resveratol-treated mice showed larger mitochondria structures and DNA content, significant increase in gene expression of pathways characteristically related to energy homeostasis, including PPARα involved β-oxidation of fatty acids and UCP1. These effects were combined with increased gene expression of SIRT1 enhancing subsequently PGC-1α activity in brown adipocytes (Lagouge et al., 2006). In this report, the authors suggested a potential contribution of SIRT1-mediated deacytelation of PGC-1α in BAT activation (Lagouge et al., 2006; Cantó and Auwerx, 2009). Similarly, Andrade et al. (2014) showed that feeding mice a standard diet plus resveratrol induced an expression of SIRT1 and UCP1 genes in BAT. Also, resveratrol effects included an increased expression of other genes, such as PTEN, which promotes EE, and Bmp7, which is known to play a central role in brown fat development and differentiation (Tseng et al., 2008).

Taken together, these results suggest that resveratrol plays a crucial role in brown adipocytes formation and activation by enhancing the AMPK–SIRT1–PGC-1α signaling pathway. It has been hypothesized that resveratrol can exert a direct stimulatory effect on SIRT1 (Price et al., 2012), whereas other data indicate an indirect activation via AMP (Wang et al., 2015a). To this regard, it has been reported that resveratrol can directly inhibit cyclic AMP (cAMP)-specific PDEs in skeletal muscle and WAT of mice leading to elevated intracellular cAMP levels. The activation of EPAC, a cAMP effector, leads to increased intracellular calcium (Ca^2+^) concentration that activates AMPK pathway and subsequently increase SIRT1 activity. However, the effect of this resveratrol-mediated by cAMP signaling pathway on brown adipogenesis remains to be elucidated (Park et al., 2012). In contrast to animal studies, there is a lack of evidence whether resveratrol can affect WAT browning or BAT activation in humans.

## Curcumin

Curcumin, also called diferuloylmethane, is a yellow-colored hydrophobic polyphenol found in extracts of Turmeric roots (a plant of the ginger family). Curcumin is commonly used as a spice in cooking and has been recognized for its potential value as an anti-obesity agent (Mantzorou et al., 2018). A recent clinical trial assessed the safety and effectiveness of 30 day treatment with curcumin combined with phosphatidylserine in overweight subjects undergoing weight loss by diet and lifestyle intervention. In this study, curcumin administration increased weight loss, enhanced the fat mass loss and induced a reduction in waist and hip circumference (Di Pierro et al., 2015).

Wang et al. (2015b) demonstrated that intra-gastric administration of curcumin (50 or 100 mg/kg daily) in mice for 50 days induced a decrease in fat mass and body weight without altering food intake. After cold challenge (exposure to 4°C), mice treated with curcumin exhibited an increase in adaptive thermogenesis, blood norepinephrine concentrations, and expression of characteristic BAT genes (e.g., ADRB3, CIDEA, PRDM 16, PGC-1α, and UCP1) in inguinal WAT (Wang et al., 2015b). In another study, Lone et al. (2016) added evidence on the effect of dietary curcumin (10–20 μM/day) in inducing a beige phenotype in white adipocytes from rats. These changes were accompanied by increased gene expression of brown fat markers such as FGF21, Tbx1, TMEM26, and CIDEA and some brown cell marker proteins including PRDM16, UCP1 and PGC-1α in a dose-dependent manner. These curcumin-induced browning effects have been shown to be mediated via the activation of AMPK-pathway (Lone et al., 2016).

In a recent study, mice fed with HFD in association with curcumin showed an increase in EE and adaptive thermogenesis following mild cold exposure. Within this study, curcumin treatment was associated with increased UCP1 expression in BAT, possibly involving PPAR-dependent and independent mechanisms (Song et al., 2018).

A common feature in these animal and human studies was the administration of high doses of curcumin. This was justified by the low systemic bioavailability of oral curcumin which also can be a reason of non-guaranteed positive results (Pan et al., 1999; Ireson et al., 2001). To this regard, Nishikawa et al. (2018) demonstrated that administration of low doses of curcumin formulations but having higher bioavailability compared to native curcumin, enhanced *in vivo* WAT-browning and increased EE in mice. These effects were induced via norepinephrine production by alternatively activated macrophages in WAT (Nishikawa et al., 2018).

## Green Tea

Green tea is a widely consumed beverage extracted from leaves of Camellia Sinensis. Several reports indicated that green tea may induce weight loss by enhancing EE and fat oxidation in humans (Westerterp-Plantenga et al., 2006). These beneficial effects are, at least in part, attributable to tea catechins such as EGCG which is the most active catechin in green tea, epigallocatechin, and epicatechin gallate (Basu and Lucas, 2007). Interestingly, green tea extracts have substantial amounts of caffeine which is known for its thermogenic properties (Westerterp-Plantenga et al., 2006). Tea catechins intake in rats fed with normal-fat diet induced an increase in BAT UCP1 expression and a loss in WAT mass (Nomura et al., 2008). Chen et al. (2017) have shown that oral administration by gavage of green tea for 8 weeks improved obesity-related parameters and upregulated the expression of BAT markers (i.e., PPAR-γ, PRDM-16, and PGC-1α) in WAT of rats fed with a HFD-diet.

With regard to human studies, Dulloo et al. (1999) demonstrated that green tea enhances EE and fat oxidation. In this study, the administration of equivalent amounts of caffeine found in green tea extracts failed to induce similar metabolic effects (Dulloo et al., 1999). However, catechins and caffeine may synergically mediate an adrenergic-induced BAT thermogenesis by acting at different check-points of the norepinephrine-cAMP axis. It was suggested that green tea catechins may promote the SNA by reducing the degradation of norepinephrine through a direct inhibition of COMT. Interestingly, caffeine may synergically prolong the effects of norepinephrine by direct inhibition of PDEs activity (Dulloo et al., 1999, 2000). The synergistic thermogenic effect exerted by EGCG and caffeine was further confirmed after administration of encapsulated EGCG-caffeine mixtures (Bérubé-Parent et al., 2005).

However, the role of green tea in tackling obesity seemed controversial in several human trials (Huang et al., 2014). It was hypothesized that these findings might be influenced by the body composition, dietary habits and ethnicity of the studied populations (Huang et al., 2014). In addition, most studies aimed to assess the impact of green tea catechins on fat oxidation rather than thermogenesis (Rains et al., 2011), therefore more studies are warranted to elucidate the role of green tea in the activation and recruitment of BAT in humans.

## Menthol

Menthol (2-isopropyl-5-methyl-cyclohexanol) also known as mint camphor, is a cyclic monoterpene alcohol produced synthetically or obtained from peppermint Mentha piperita (Patel et al., 2007; Pergolizzi et al., 2018). For centuries, menthol has found application in medical field due to its promising biological properties including antitussive, anti-inflammatory, antipruritic, antibacterial and analgesic effects (Patel et al., 2007). Menthol is also known to induce cooling sensation by activating the TRPM8 receptor, a Ca^2+^-permeable non-selective channel that detects cold stimuli in the thermosensory system (McKemy et al., 2002; Bautista et al., 2007). TRPM8 is also expressed in significant amounts in the lungs, male reproductive system and cancerous tissues where its function is yet not well understood (Tsavaler et al., 2001; Stein et al., 2004). Recent studies have demonstrated TRPM8 expression on the membrane of brown and white adipocytes (Ma et al., 2012; Rossato et al., 2014; Jiang et al., 2017).

Long-term administration of menthol in mice has been shown to enhance UCP1 expression and activity in BAT, increase EE, ameliorate insulin sensitivity and prevent HFD-induced weight gain. These effects have shown to be mediated by TRPM8 activation in brown adipocytes with consequent Ca^2+^-dependent PKA phosphorylation (Ma et al., 2012). Similarly, Jiang et al. (2017) reported that TRPM8 is also expressed on cultured white adipocytes from mice and its activation by menthol enhanced the expression of thermogenic genes (UCP1, PGC1α) via PKA signaling pathway in white adipocytes. Interestingly, menthol administration protected mice against HFD-obesity and enhanced beige adipocytes (Jiang et al., 2017).

In addition, TRPM8 activation *in vitro* by menthol in human white adipocytes induced a brown-like phenotype, stimulated UCP1 expression and adipocyte thermogenesis (Rossato et al., 2014). Moreover, topical application of menthol on skin has been shown to activate TRPM8 and induce parallel increase in NST and body temperature (Tajino et al., 2007; Vizin et al., 2018).

Taken together, these findings reinforce evidence about the prospective use of menthol as a promising approach in the management of obesity and associated comorbidities by regulating energy balance and metabolic homeostasis.

## Fish-Derived Omega-3 Fatty Acids

Omega-3 PUFAs such as DHA and EPA are major polyunsaturated fats found fish oil supplements and in fatty fish such as salmon (Anderson and Ma, 2009). The supplementation of fish oil had shown to increase UCP1 expression (Takahashi and Ide, 2000) and protein levels in interscapular BAT of rats (Kawada et al., 1998). Recently, dietary n-3 PUFAs has shown to lower the amount of visceral WAT and increase the mass of interscapular BAT in rats (Crescenzo et al., 2017).

It has been shown that fish oil increases the expression of numerous thermogenic genes in BAT including β3 ADRB3, PRDM16, PGC1α and PPARs in BAT (Bargut et al., 2016; Pahlavani et al., 2017). In addition, in inguinal WAT fish oil exerts its browning effects by recruiting beige adipocytes. In another study, Kim et al. (2015) added evidence that fish oil supplementation in rats for 10 weeks induced a recruitment of beige adipocytes by increasing the expression of UCP1, PGC1α, CIDEA and PRDM16 in inguinal WAT. The authors suggested that fish oil contributes to brown adipogenesis by acting as ligand of TRPV1 in digestive tract that triggers, via the brain, a β2-adrenergical sympathetic response in adipose depots.

Furthermore, Laiglesia et al. (2016) have recently shown that EPA induces a switch from white to beige-like adipocytes in human subcutaneous adipocytes of overweight subjects by promoting the activation of AMPK/SIRT1/PGC1- α axis. However, despite promising data from murine studies, little is known about the thermogenic activity of n-3 PUFAs in humans.

## Conclusion

The recent discovery of metabolically active BAT in adult humans has raised the expectations for the development of novel anti-obesity treatments that can regulate brown or beige fat development. In this review we focused on few dietary molecules that have shown to regulate BAT activation or beige fat development. Despite the promising data from animal models or cell lines, these findings need to be validated in humans by further large clinical trials with relatively long-term period of follow-up and taking in consideration factors such as ethnicity, genetics and lifestyles. Moreover, current knowledge deriving from cell culture and animal models suggests that polyphenols, mainly curcumin, and resveratrol, exert their thermogenic effect when supplemented at doses that are quite elevated. Therefore, further research is warranted to define the optimal preparation, doses as well as the bioavailability and safety of these molecules in humans.

Finally, the above discussed dietary components have been shown to share common molecular targets involved in the induction of brown adipogenesis. Then future studies should test the hypothesis whether combined supplementations may also operate synergistically to activate NST in human through activation of BAT and/or beige adipose tissue recruitment.

## Author Contributions

HE wrote the manuscript. MR proofread the manuscript and provided guidance on the overall direction of the manuscript. All authors critically appraised the final version of the paper.

## Conflict of Interest Statement

The authors declare that the research was conducted in the absence of any commercial or financial relationships that could be construed as a potential conflict of interest.

## Abbreviations

ADRB3: β3-adrenergic receptor
AMPK: 5′adenosine monophosphate-activated protein kinase
ATP: adenosine triphosphate
BAT: brown adipose tissue
BMP8B: bone morphogenetic protein 8b
C/EBP-α: CCAAT-enhancer binding protein alpha
cAMP: cyclic adenosine monophosphate
CD137: member of tumor necrosis factor receptor
CIDEA: cell death-inducing DFFA-like effector a
COMT: catechol-O-methyl-transferase
CVDs: cardiovascular diseases
DHA: docosahexaenoic acid
EE: energy expenditure
EGCG: epigallocatechin gallate
EPA: eicosapentaenoic acid
EPAC: exchange protein directly activated by cAMP
FAS: Death receptor
FDG-PET [^18^F]: fluorodeoxyglucose–positron emission tomography
FGF21: fibroblast growth factor 21
HFD: high-fat diet
HSL: hormone-sensitive lipase
LPL: lipoprotein lipase
Mfn2: mitofusin 2
mRNA: messenger RNA
NAD+: nicotinamide adenine dinucleotide
NST: non-shivering thermogenesis
PDEs: phosphodiesterases
PGC-1α: peroxisome proliferator-activated receptor gamma coactivator 1-alpha
PKA: protein kinase A
PPARγ: peroxisome proliferator-activated receptor gamma
PRDM16: PR-domain containing 16
PTEN: phosphatase and tensin homolog
PUFAs: polyunsaturated fatty acids
SIRT1: sirtuin-1
SNA: sympathetic nerve activity
SREBP-1c: sterol regulatory element binding protein-1c
Tbx1: T-box transcription factor 1
TMEM26: transmembrane protein 26
TRPM8: transient receptor potential cation channel melastatin 8
TRPV1: transient receptor potential vanilloid 1
UCP1: uncoupling protein 1
VO_2_: oxygen consumption
WAT: white adipose tissue
